# Enhanced Photocatalytic Degradation of Ternary Dyes by Copper Sulfide Nanoparticles

**DOI:** 10.3390/nano11082000

**Published:** 2021-08-04

**Authors:** Peter A. Ajibade, Abimbola E. Oluwalana

**Affiliations:** School of Chemistry and Physics, University of KwaZulu-Natal, Private Bag X01, Scottsville, Pietermaritzburg 3209, South Africa; 217075609@stu.ukzn.ac.za

**Keywords:** copper sulfide, nanoparticle, ternary dye, photocatalytic degradation, adsorption kinetic

## Abstract

We report the effect of thermolysis time on the morphological and optical properties of CuS nanoparticles prepared from Cu(II) dithiocarbamate single-source precursor. The as-prepared copper sulfide nanoparticles were used as photocatalysts for the degradation of crystal violet (CV), methylene blue (MB), rhodamine B (RhB), and a ternary mixture of the three dyes (CV/MB/RhB). Powder XRD patterns confirmed the hexagonal covellite phase for the CuS nanoparticles. At the same time, HRTEM images revealed mixed shapes with a particle size of 31.47 nm for CuS**1** prepared at 30 min while CuS**2** prepared at 1 h consists of mixtures of hexagonal and nanorods shaped particles with an average size of 21.59 nm. Mixed hexagonal and spherically shaped particles with a size of 17.77 nm were obtained for CuS**3** prepared at 2 h. The optical bandgaps of the nanoparticles are 3.00 eV for CuS**1**, 3.26 eV for CuS**2** and 3.13 eV for CuS**3.** The photocatalytic degradation efficiency showed that CuS**3** with the smallest particle size is the most efficient photocatalyst and degraded 85% of CV, 100% of MB, and 81% of RhB. The as-prepared CuS showed good stability and recyclability and also degraded ternary dyes mixture (CV/MB/RhB) effectively. The byproducts of the dye degradation were evaluated using ESI-mass spectrometry.

## 1. Introduction

Rapid growth in industrial activities in the past decades has resulted in the continuous discharge of heavy metals and toxic organic pollutants into water bodies. The presence of these contaminants poses great dangers to the aquatic ecosystem and human health. Methods such as ozonation, biochemical treatment, and electrochemical oxidation have been utilized to remove these pollutants but are hindered by several limitations [1,2,3]. Their limitation led to the development of advanced oxidation processes (AOPs), including Fenton, photo-Fenton, and photocatalysis [4,5,6]. Among these, photocatalysis that involves the generation of reactive oxidative species for the degradation of organic pollutants is used more frequently. TiO_2_ has been widely used as a photocatalyst, but its use is hindered due to its large energy bandgap, limiting light absorption to the UV region [7]. Thus, it is imperative to develop semiconductors nanoparticles with enhanced photocatalytic efficiency. The use of copper sulfide nanoparticles as an efficient catalyst has received attention catalyst for dye degradation due to its low bandgap energy that enables it to absorb light through a wide spectrum [8]. CuS exists in different crystalline phases such as CuS (covellite), Cu_7_S_4_-CuS (hexagonal plates), Cu_1.75_S (anilite), Cu_2_S (chalcocite), Cu_9_S_5_ (digenite octahedron) [9]. CuS in the covellite crystalline phase has a direct bandgap of 1.2–2.0 eV due to its low and high reflectance in the visible and near-infrared regions [10], which makes it a good semiconductor for solar cell applications [11], biosensors [12], gas sensors [13], energy storage [14], and photocatalysis [15].

Copper sulfide is being explored as a photocatalyst because it is environmentally friendly, cheap, non-toxic, easy to regenerate, biocompatible, great chemical stability, unique optical and electrical properties, enhancing its effectiveness for the removal of dyes from wastewater [9,16,17]. Isac et al. [18] reported 84% degradation of methylene blue after 4 h under visible light by CuS. Zhang et al. [19] reported Cu_2-x_S-diatomite that effectively photodegraded 99.1% and 96.9% methylene blue and methyl orange respectively in H_2_O_2_, under UV-Vis light irradiation. Borkthakur et al. [20] prepared CuS nanoparticles by microwave-assisted synthesis, which degraded 59% congo red dye under natural sunlight irradiation. Previous studies have shown that the composition, surface morphology, and crystalline phase of CuS nanoparticles influence their photocatalytic efficiencies [21,22]. These properties are affected by the synthetic methods used for the preparation of CuS nanoparticles, which includes solvothermal [23], hydrothermal [24], sol-gel [25], micro-emulsion [26], microwave [27], sonochemical [28], electrospinning [29] and single-source precursors [30,31]. The use of a single-source precursor to prepare CuS nanoparticles has been reported to give high-quality nanoparticles due to the metal-sulfur bond in the precursors [32,33].

In this study, we present the effect of thermolysis time on the structural and optical properties of CuS nanoparticles Cu(II) dithiocarbamates complex and the use of the CuS as a nanophotocatalyst to effectively degrade crystal violet (CV), methylene blue (MB), rhodamine B (RhB) and ternary dyes (CV/MB/RhB) mixture. To the best of our knowledge, the photocatalytic degradation of ternary dyes mixture by CuS has not been reported. Hence we studied the simultaneous removal of toxic ternary dyes over CuS, which can be developed further for potential industrial applications to remove different dyes. The effects of catalytic dosage, irradiation time, pH, and scavenging ability on the photocatalytic process were investigated. In addition, the stability and reusability of the as-prepared CuS nano photocatalyst were also evaluated.

## 2. Materials and Methods

### 2.1. Materials

Oleic acid (99%), octadecylamine (90%), methanol (≥99.9%), toluene (≥99.5%), crystal violet (≥90.0%), methylene blue, rhodamine B (95%), sodium sulphate (≥99.0%), sodium oxalate (≥99.5%), acrylamide (98%) and isopropanol were purchased from Merck (Darmstadt, Germany). All the reagents were used as purchased without further purification. Copper(II) morpholine dithiocarbamate was prepared using the reported procedure [34].

### 2.2. Synthesis of CuS Nanoparticles

We dissolved 3 mmol of copper(II) morpholine dithiocarbamate in 30 mmol oleic acid, and the slurry obtained was injected into hot octadecylamine (30 mmol) at 130 °C. A decrease of temperature to 117 °C was observed. The temperature was stabilized at 130 °C and maintained for the appropriate time (30 min, 1 h, 2 h) under nitrogen flow while stirring. The reaction mixture was cooled to 70 °C, and methanol was added to precipitate the nanoparticles. The nanoparticles were isolated by centrifugation and rinsed with methanol to remove excess octadecylamine. The resulting solid was dried at room temperature. The copper sulfide nanoparticles were labeled CuS**1** (30 min), CuS**2** (1 h), and CuS**3** (2 h).

### 2.3. Characterization Techniques

The crystal phase of the samples was identified by powder X-ray diffraction using Bruker D8 advanced diffractometer (Billerica, MA, USA) Cu-Kα irradiation (λ = 1.5405 Å). The microstructure nature of the samples was characterized with the use of a high-resolution transmission electron microscope (HRTEM, JEOL-2100, (Akishima, Tokyo, Japan)). A scanning electron microscope (ZEISS FEGSEM, Oberkochen, Germany) equipped with electron diffraction spectroscopy at 20 kV rating voltage was used for the morphology and elemental composition of the CuS nanoparticles. The room temperature photoluminescence was measured using Perkin-Elmer (Waltham, MA, USA) LS 45 fluorescence spectrometer and UV-Visible absorption spectra were measured using Perkin-Elmer Lambda 25 UV-Vis spectrophotometer (Waltham, MA, USA).

### 2.4. Adsorption Studies of CuS Nanoparticles

Methylene blue, crystal violet, rhodamine B, and the mixed dye adsorption studies were evaluated using CuS**1**. The experiment was done by varying the catalyst dosage (20, 40, 40, 60, 80, and 100 mg) in 50 mL of aqueous dye solution in a dark room. An aliquot was taken at 15 min intervals for 75 min and monitored by a UV-Visible spectrometer after centrifugation. The adsorption capacity at equilibrium (*Q_e_*) and time ‘*t*’ (*Q_t_*) are represented using Equations (1) and (2) [35].

(1) $$Q_{e}=\left(\frac{C_{o}-C_{e}}{W}\right)V$$

(2) $$Q_{t}=\left(\frac{C_{o}-C_{t}}{W}\right)V$$

where *C_o_*, *C_e_*, *C_t_* (mg/L), *W,* and *V* are the initial dye concentration, equilibrium concentration, concentration at a specific time, weight of the adsorbent, and volume of dye, respectively.

### 2.5. Photocatalytic Experiment

The photocatalytic activity was evaluated by monitoring the degradation of the organic dyes (crystal violet, methylene blue, and rhodamine B) under visible light irradiation. Then, 80 mg of the catalyst was dispersed in 50 mL of the aqueous solution (5 ppm). The mixture was subsequently stirred well to obtain a homogenous mixture and afterward stirred in the dark for 45 min to attain adsorption-desorption equilibrium. The mixture was then exposed to visible light irradiation (70 W mercury lamp). An aliquot was taken at 20 min intervals for 120 min. The pH of the dyes was adjusted by the addition of 0.1 M HCl or 0.1 M NaOH solution.

### 2.6. Photocatalytic Degradation of CV/MB/RhB Dyes Mixture

An aqueous solution of CV/MB/RhB dyes mixtures were prepared (5 ppm). 80 mg of as-prepared CuS nanoparticles were added into 50 mL of dyes mixture. The suspension was stirred in a dark condition for 45 min. Afterward, the suspension was exposed to mercury lamp visible light (70 W). A UV-visible spectrophotometer was used to measure the absorbance of the photo irradiated suspension. The initial dyes and degraded intermediate products were detected through Shimadzu LCMS-2020 mass spectrometer (Kyoto, Japan).

### 2.7. Determination of Oxidative Reactive Species

The main reaction was quenched with the use of three scavengers: Acrylamide (AC) superoxide scavenger, sodium sulfate (NaS) electron scavenger, sodium oxalate (NaOx) hole scavenger, isopropanol (IPA), hydroxyl radical scavenger.

## 3. Results and Discussion

**Figure 1:**
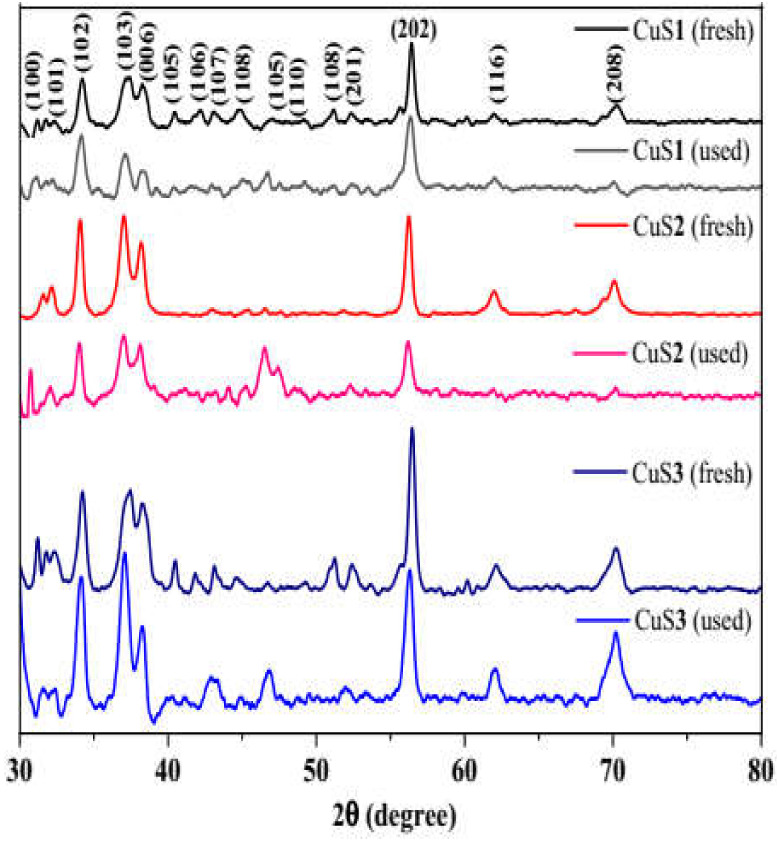
Comparison of XRD patterns of CuS nanoparticles for fresh and after photocatalysis (after four cycles).

Powder XRD patterns of fresh CuS and used CuS nanoparticles after photocatalysis were compared (Figure 1) to establish any changes in the crystalline phase of the as-prepared CuS nanoparticles. The CuS nanoparticles showed diffraction peaks at 2θ = 31.04°, 31.67°, 34.16°, 37.17°, 38.18°, 40.43°, 41.77°, 43.05°, 44.83°, 46.96°, 49.03°, 51.08°, 52.27°, 56.38°, 61.94° and 70.19° which corresponds to (100), (101), (102), (103), (006), (105), (106), (107), (008), (105), (110), (108), (201), (202), (116) and (208) plane of hexagonal covellite CuS phase (JCPDS No: 01-078-0876) [36,37]. The diffraction peaks show no apparent deviations; after the CuS nanoparticles have been used as photocatalysts to degrade the organic dyes. It is worth noting that the diffraction patterns of CuS**3** prepared at 2 h are well defined. No peaks in the XRD could be attributed to other CuS phases. This indicates that the thermolysis of the Cu(II) dithiocarbamate complex resulted in a pure hexagonal covellite CuS phase.

**Figure 2:**
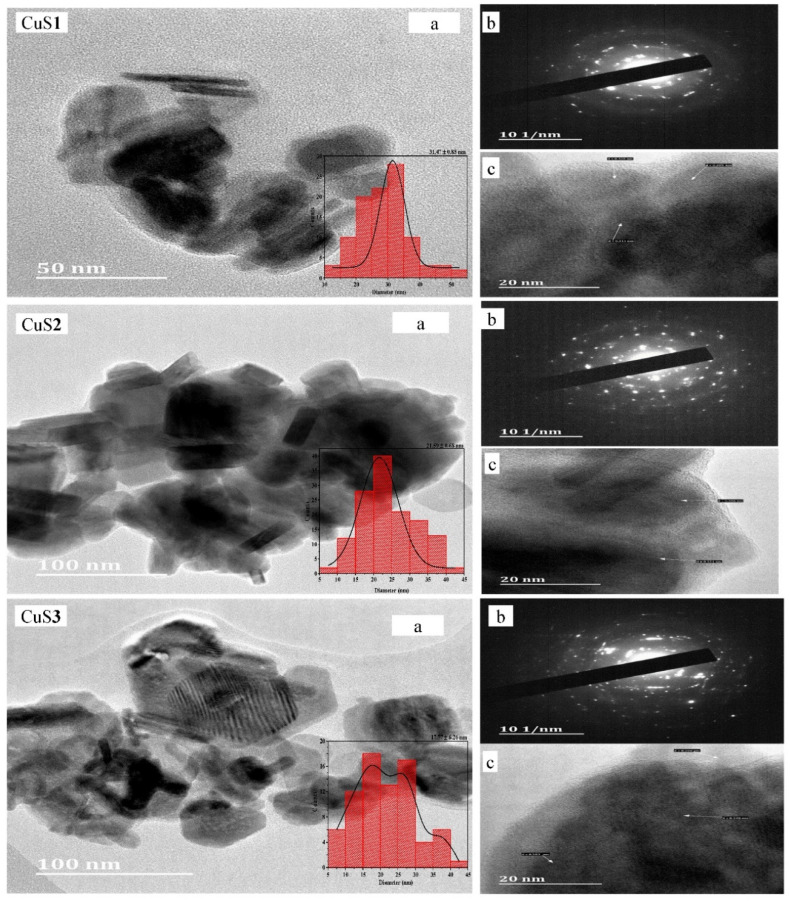
HRTEM images (**a**) SAED (**b**) and lattice fringes (**c**) and corresponding size distribution chart (insert) of CuS**1**, CuS**2** and CuS**3** nanoparticles.

HRTEM images of the as-prepared CuS nanoparticles are presented in Figure 2a. CuS**1** is a mixture of spherical, triangular, hexagonal, and nanorods with an average particle size of 31.47 ± 0.85 nm while CuS2 consists of hexagonal and nanorods with an average size of 21.59 ± 0.68 nm. CuS**3** is a mixture of hexagonal nanorods and spherically shaped nanoparticles with an average size of 17.77 ± 6.26 nm. The HRTEM images revealed that CuS with smaller particles sizes were obtained at 2 h due to the Oswald ripening process because of the longer reaction time [38]. The agglomeration observed in the HRTEM images of CuS**1** and CuS**2** could be due to high surface energy [39]. The results suggest that the thermolysis time influences the shapes and particle sizes of the as-prepared CuS nanoparticles.

### 3.1. Structural and Morphological Studies of the CuS Nanoparticles

The selected area electron diffraction (SAED) of the as-prepared CuS nanoparticles (Figure 2b) showed a brightly dispersed dotted pattern which is attributed to the single-crystalline nature of the nanoparticles [40]. However, the scattered spots indicate that the nanoparticles are not monodispersed [41]. The lattice fringes in Figure 2c shows that CuS**1** has interplanar *d* spacing of 0.308 nm and 0.309 nm, which corresponds to (102) plane, while CuS**2** interplanar spacing is 0.321 nm and 0.306 nm, which are consistent with (101) and (102) plane of covellite CuS. CuS**3** yielded *d*-spacing of 0.308 nm, 0.320 nm, and 0.261 nm corresponding to (102), (101), and (006) planes of covellite CuS [42,43].

SEM images shown in Figure S1 revealed the as-prepared CuS nanoparticles have different surface morphologies. The morphologies varied from flowery to irregular spheres as thermolysis time increased from 30 min to 2 h. EDX spectra analysis of the CuS nanoparticles confirmed the atomic ratio of Cu and S to be approximately 1:1. The carbon and oxygen peaks are ascribed to the capping agents used, while the gold peak is due to the coating used for SEM/EDX analysis. The elemental mapping of the CuS nanoparticles showed homogenous distributions of copper and sulfur in the mapped area.

The FTIR spectra of octadecylamine (capping agent) and the as-prepared CuS nanoparticles (Figure S2) showed three peaks at 3322, 3265, and 3181 cm^−1^ assigned to υ(N–H) stretching vibrations while the υ(C–H) symmetric and asymmetric vibrations were observed at 2909 and 2842 cm^−1^ [44]. The υ(N–H) peaks shifted to 3317, 3227, and 3054 cm^−1^ in the octadecylamine capped CuS nanoparticles while the υ(C–H) symmetric vibrations shifted to 2911 cm^−1^ and υ(C–H) asymmetric vibration was observed at 2838 cm^−1^. The observed shifts in the υ(N–H) vibrations indicate that the as-prepared CuS nanoparticles were stabilized by octadecylamine on the nanoparticles through –NH_2_ [45]. Thus, the FTIR spectra confirmed that octadecylamine is strongly adsorbed on the surface of the as-prepared CuS nanoparticles.

### 3.2. Optical Studies of the CuS Nanoparticles

**Figure 3:**
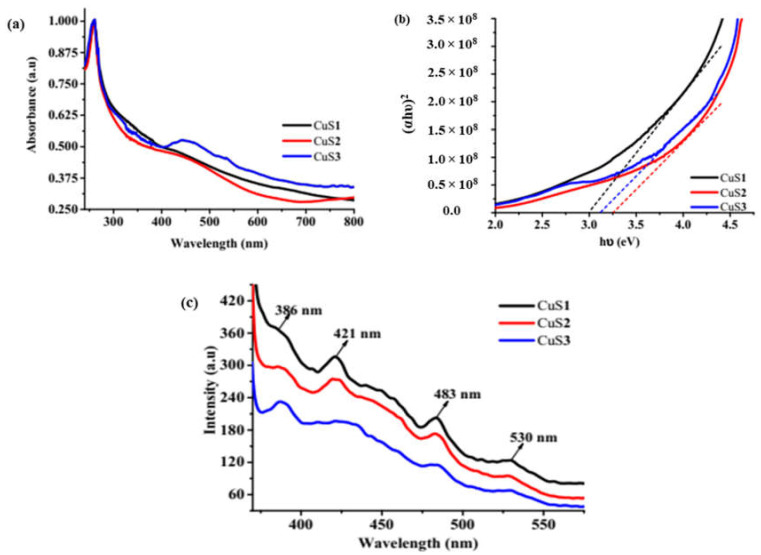
Ultraviolet-visible absorption spectra (**a**) Tauc’s plot (**b**) and photoluminescence spectra (**c**) of as-prepared CuS nanoparticles.

The absorption of the as-prepared CuS nanoparticles was studied using UV-Vis spectroscopy (Figure 3a). The absorption bands at 259 and 452 nm are characteristics of covellite CuS [46]. The absorption of the CuS nanoparticles from the ultraviolet to the near-infrared region is due to the color of the nanoparticles [37].

The as-prepared CuS nanoparticles’ optical bandgap was determined using the Tauc plot by plotting (αhυ)^2^ against (hυ) and extrapolating the slope of the band edge against zero as shown in Figure 3b. The estimated optical bandgaps are 3.00 eV, 3.26 eV and 3.13 eV for CuS**1**, CuS**2** and CuS**3** respectively. The estimated energy bandgaps are greater than that of bulk CuS (1.2 eV). This indicates that the as-prepared CuS nanoparticle’s absorption band edges are blue-shifted due to the quantum confinement effect due to the nanoparticle’s particles sizes [41]. The recombination and transfer of photo-induced electrons and holes were studied using photoluminescence spectroscopy [47]. Figure 3c shows the room temperature photoluminescence spectra for the CuS nanoparticles excited at 350 nm. The spectra showed broad emission peaks at 385 nm, 421 nm, 483 nm, and 530 nm. The peak at 385 nm is ascribed to electron-hole recombination in the surface states [48]. The emission peaks in the visible region (421 and 483 nm) are attributed to surface defects [49]. The green emission band at around 530 nm is ascribed to radiative recombination between the conduction band and copper vacancy-related acceptor levels around the valence band edge [48]. The emission peaks observed in this study are consistent with the literature [47,49,50]. The slight shift observed in the photoluminescence spectra of the CuS nanoparticles might be due to different particle sizes and morphology.

### 3.3. Adsorption Studies

**Figure 4:**
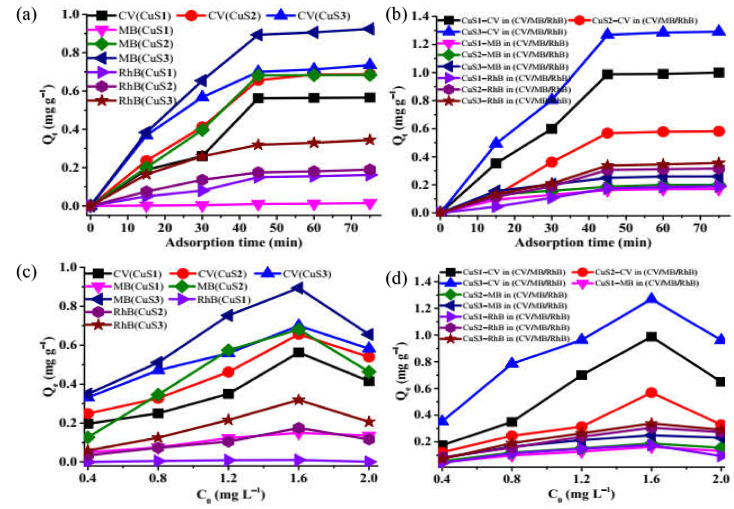
Time profile adsorption capacity (**a**,**b**) and catalytic dosage adsorption capacity (**c**,**d**) of CuS over single and mixed dyes.

The CuS nano photocatalyst reached equilibrium adsorption capacity in the photocatalytic process at 45 min for all the dyes. Above 45 min, the adsorption capacity was stable, which indicates the attainment of adsorption-desorption equilibrium. Figure 4c,d shows that the dyes adsorb on the catalyst surface strongly in the range of 0.4 mgL^−1^–1.6 mgL^−1^. An increase in catalyst dosage from 1.6 mgL^−1^ to 2 mgL^−1^ resulted in a reduction of adsorption capacity in the dyes, which might be due to adsorbent particle aggregation [51].

### 3.4. Effect of Catalyst Dosage

**Figure 5:**
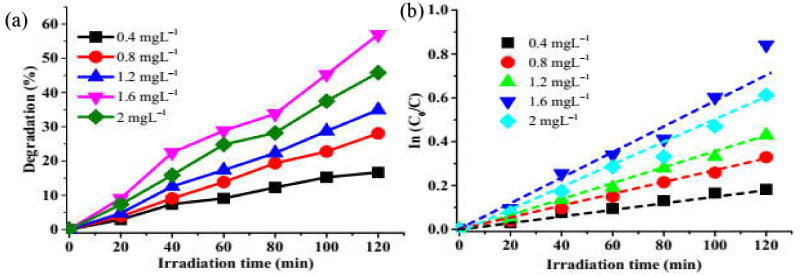
Effect of CuS**1** catalytic dosage against crystal violet (**a**) and the corresponding plot of In(C_0_/C) versus irradiation time (**b**).

Optimization of CuS**1** catalyst dosage (0.4 mgL^−1^–2 mgL^−1^) was performed using crystal violet. The graph in Figure 5a shows that the degradation efficiency of 56.9% was obtained at 1.6 mgL^−1^, decreasing to 45.8% with 2 mgL^−1^ of the catalyst. This might be due to the turbidity of the catalyst suspension as the concentration increases, which reduces the penetration of light through the dye, hindering the catalyst activation [52]. The kinetic modeling result is shown in Figure 5b. The rate constants obtained are 0.0016, 0.0028, 0.0038, 0.0066, 0.005 min^−1^ with R^2^ values of 0.9899, 0.996, 0.9869, 0.9674 and 0.9859 for 0.4 mgL^−1^, 1.8 mgL^−1^, 1.2 mgL^−1^, 1.6 mgL^−1^ and 2 mgL^−1^ respectively which shows that the degradation process follows a pseudo-first-order kinetics. The concentration of dyes was kept constant throughout the experiments with the optimized conditions of all CuS samples photodegradation attributes evaluated against crystal violet. The use of a 1.6 mgL^−1^ catalyst dosage degraded CV four times faster than 0.4 mgL^−1^, indicating that increase in catalyst dosage increases the degradation efficiency. All the R^2^ values were greater than 0.9674, confirming that the pseudo-first-order model is a reliable and appropriate model to study the photodegradation process [53].

### 3.5. Photocatalytic Degradation of Crystal Violet (CV), Methylene Blue (MB), Rhodamine B, and CV/MB/RhB by the CuS Nanoparticles

**Figure 6:**
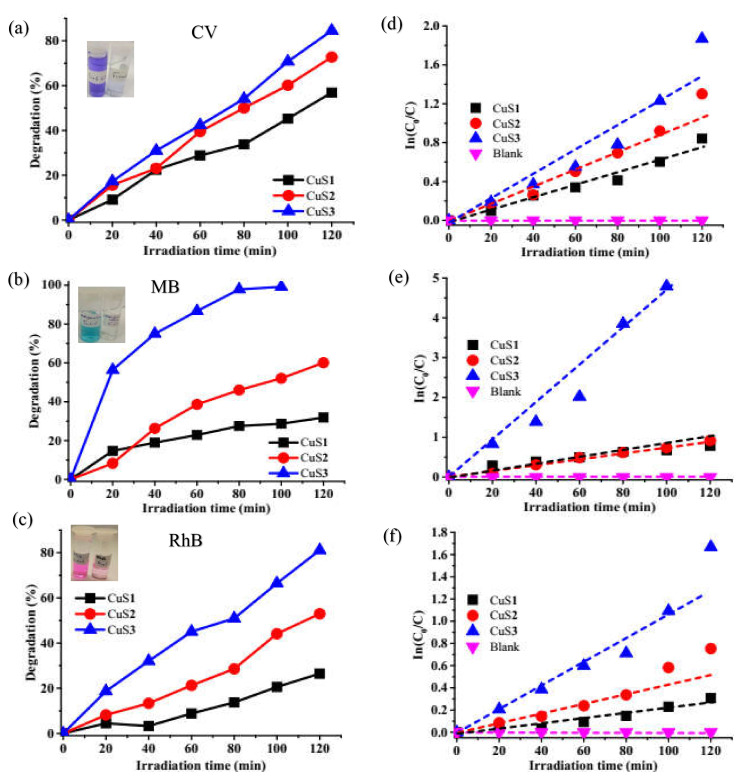
Degradation curve of organic dyes using CuS nanoparticles (**a**–**c**) and the corresponding plot of In(C_0_/C) versus irradiation time (**d**–**f**).

The as-prepared CuS nanoparticles’ photocatalytic potential was studied against single dyes solution of CV, MB, RhB, and ternary mixture of the three dyes (CV/MB/RhB) under visible light irradiation (Figures S3–S5). The dyes absorption maxima decrease with an increase in visible light irradiation. The degradation efficiencies of CuS**1**, CuS**2,** and CuS**3** against CV were 56.9%, 72.8%, and 84.6%, respectively. Meanwhile, against MB it was 31.8%, 60.1% for CuS**1** and CuS**2** after 180 min, and 100% for CuS**3** after 100 min (Figure 6). RhB, another common cationic dye, was also studied under the same conditions, and degradation efficiencies of 26.5%, 53%, and 81.4% were obtained for CuS**1**, CuS**2,** and CuS**3,** respectively. The results showed that CuS**3** is a highly efficient catalyst for the degradation of CV (84%), MB (100%), and RhB (81%). The enhanced degradation efficiency of CuS**3** might be due to its smaller particle size and almost uniform size distributions. The intense color of the starting dye solution faded gradually during the photodegradation process as exposure time increases (insert Figure 6). The effectiveness of CuS nanoparticles is ascribed to their surface defects and Cu-vacancies that exist on the surface of CuS [54]. In addition, a comparison of the degradation efficiency of the CuS nanoparticles with their respective sizes shows that the percentage degradation increases with a decrease in particle size. The recombination of electron-holes on a large surface is high and subsequently reduces the availability of free charges on the surface, thereby reducing photocatalytic efficiency [55]. As stated earlier from the rate of electron-hole recombination observed in the photoluminescence spectra, CuS**3** was observed to be low. This increases the surface area of the nanoparticles to participate in the photodegradation process [55]. In this study, a shorter degradation time was achieved using a light source with lower wattage. Hence, the as-prepared copper sulfide showed better degradation efficiency for organic dyes when compared with previous studies, as presented in Table 1.

**Figure 7:**
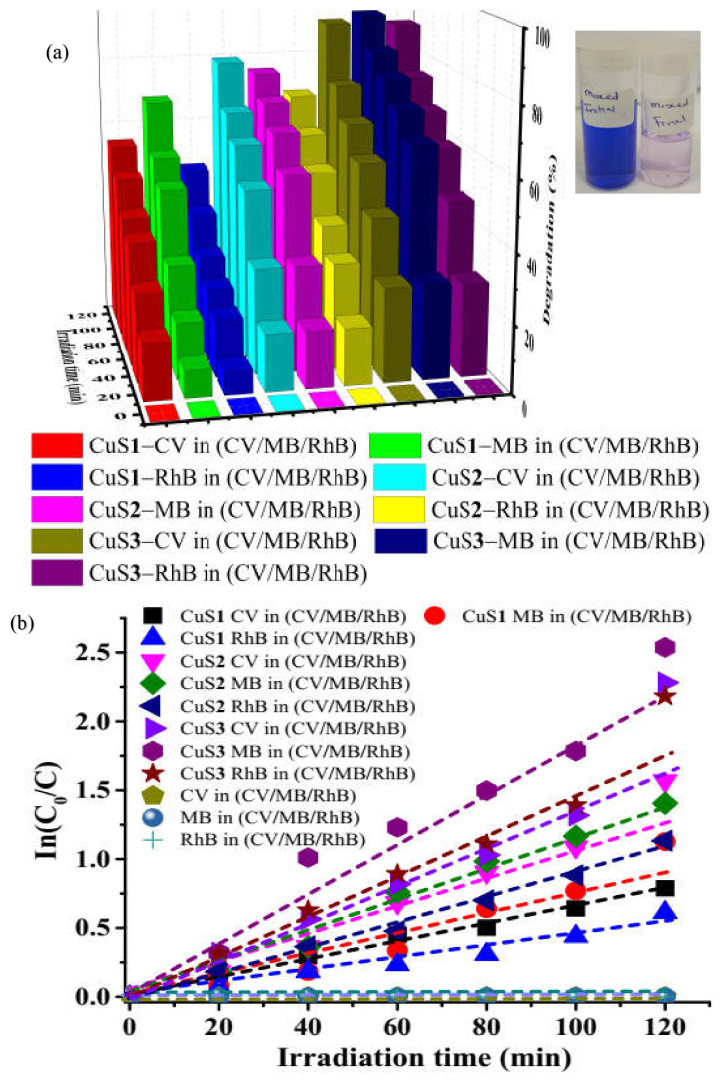
Degradation efficiency bar chart of ternary dyes (mixture of crystal violet, methylene blue, and rhodamine B) (**a**) and kinetics plot (**b**) using CuS nanoparticles.

The ternary solution of crystal violet (CV), methylene blue (MB), and rhodamine B absorption spectra show no spectrum overlap between these compounds. The absorption bands at 663, 589, and 555 nm are characteristics of MB, CV, and RhB dyes used. Figures S6–S8 show the UV-absorption spectra of the mixed dye degradation with respect to time. The degradation of the individual dyes in the mixture was found to be RhB (45.8%) < CV (54.5%) < MB (67.6%) for CuS**1** while CuS**2** was RhB (67.8%) < MB (75.8%) < CV (79.2%) and CuS**3** is RhB (88.7%) < CV (89.8%) < MB (92.1%) as presented in Figure 7. Overall, CuS**3** showed superior photocatalytic activity for mixed dye compared to CuS**1** and CuS**2**. The single and mixed dye rate degradation constant was calculated using a pseudo-first-order kinetic equation, and the results are presented in Table 2. The slow rate of rhodamine B degradation compared to methylene blue and crystal violet could be attributed to its complex structure [64].

### 3.6. Scavenging Activity to Determine Mechanism of Photocatalytic Activity

**Scheme 1:**
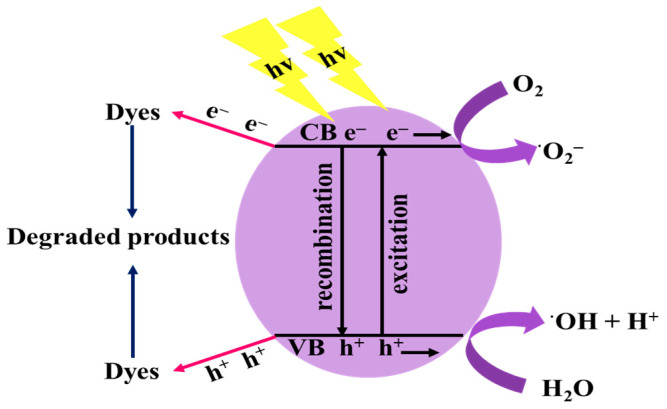
Schematic illustration of the photocatalytic degradation mechanism.

**Figure 8:**
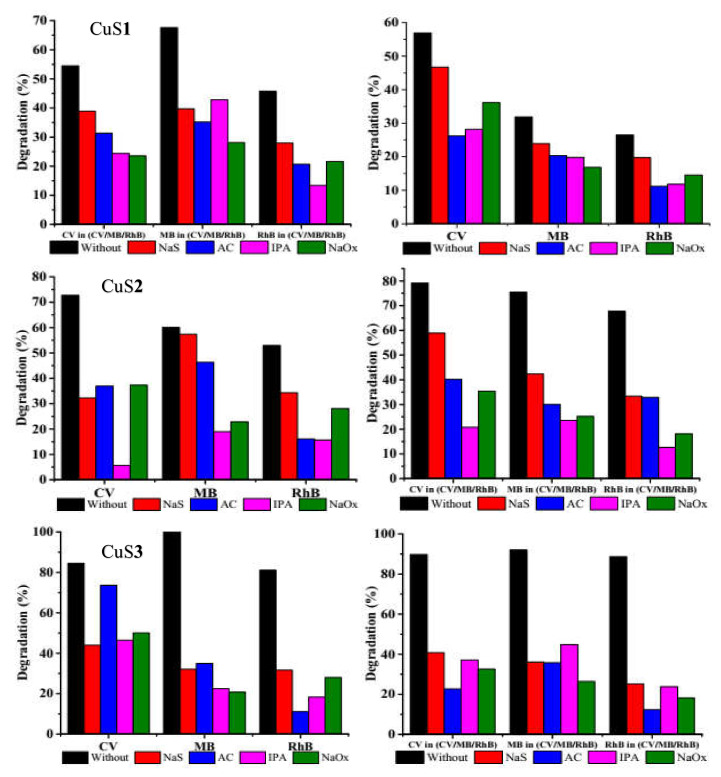
Effects of scavengers on single and ternary dyes over CuS nanoparticles.

Three different scavengers were used to investigate the photocatalytic degradation mechanism of the as-prepared CuS. Sodium sulfate (NaS, e^−^ scavenger), sodium oxalate (NaOx h^+^ scavenger), acrylamide (AC, ^•^O_2_^−^ scavenger), and isopropanol (IPA, ^•^OH scavenger) [65]. Figure 8 shows the scavenging activity of the as-prepared CuS nanoparticles over each dye and mixed dyes. The addition of NaS reduced photocatalytic degradation of CV from 56.9% to 46.7%, MB from 31.9% to 23.9%, and RhB from 26.5% to 19.8%. The reduction in the photocatalytic degradation of (CV/MB/RhB) mixed dye was 54.5% to 38.9% for CV, 67.6% to 39.7% for MB, 45.8% to 27.9% for RhB over CuS**1**. The results show that e^−^ does not play any significant role in the photocatalytic degradation of the dyes. Addition of AC to the dyes solution also led to a decrease in the photocatalytic degradation efficiency to 26.2%, 20.3%, 11.2%, 31.8%, 35.2% and 20.7% in CV, MB, RhB, CV in (CV/MB/RhB), MB in (CV/MB/RhB) and RhB in (CV/MB/RhB), respectively, which implies that ^•^O_2_^−^ is an active species. The degradation rate over CuS**1** reduced significantly in the presence of isopropanol to 28.2%, 19.8%, 11.8%, 24.4%, 42.8%, and 13.4%. The results suggest that ^•^OH was a dominant active species in the photodegradation of CV, MB, RhB, CV in (CV/MB/RhB), MB in (CV/MB/RhB), and RhB in (CV/MB/RhB). The mixture of NaOx as a hole trapping agent with the dye solutions resulted in a significant decrease to 36.16% for CV, 16.84% for MB, 15.55% for RhB, 28.60% for CV in (CV/MB/RhB), 28.14% for MB in (CV/MB/RhB) and 21.62% for RhB in (CV/MB/RhB). This suggests that h^+^ is a dominant reactive species in the degradation of the dye solutions. The photodegradation efficiency of single dye and mixed ternary dyes solution over CuS**2** and CuS**3** showed a significant decrease in the presence of IPA, AC, and NaOx. In contrast, a slight decrease was observed with NaS, which confirms that ^•^O_2_^−^, ^•^OH and h^+^ plays a significant role and e^−^ acts as a minor active species in the photodegradation process [66,67]. The photocatalytic reaction Equations (3)–(6) may occur during the degradation of dyes over CuS nanoparticles which is illustrated in Scheme 1:

(3) $$\mathrm{CuS}+\mathrm{hv}\;\to \;h^{+}\left(\mathrm{CuS}\right)+e^{-}\left(\mathrm{CuS}\right)$$

(4) $$e^{-}+O_{2}\left(\mathrm{adsorbed}\right)\;\to \;O_{2}^{•-}$$

(5) h++H2O → OH•+H+

(6) O2•−+h+/OH•dyes→→→Intermediates→CO2+H2O

The photoexcited electrons and holes are generated using the optical energy under the visible light irradiation in the conduction and valence band of the CuS nanoparticles (Equation (3)) [19]. The photogenerated holes are responsible for the oxidation of the dyes into non-toxic inorganic products by reacting with H_2_O to form ^•^OH. In contrast, e^−^ reacts with ^•^O_2_^−^ in the air to form (superoxide radical). The generated ^•^OH and ^•^O_2_^−^ radical directly react with the dyes oxidizing them into smaller molecules [65].

### 3.7. Photostability Studies of CuS nanoparticles

**Figure 9:**
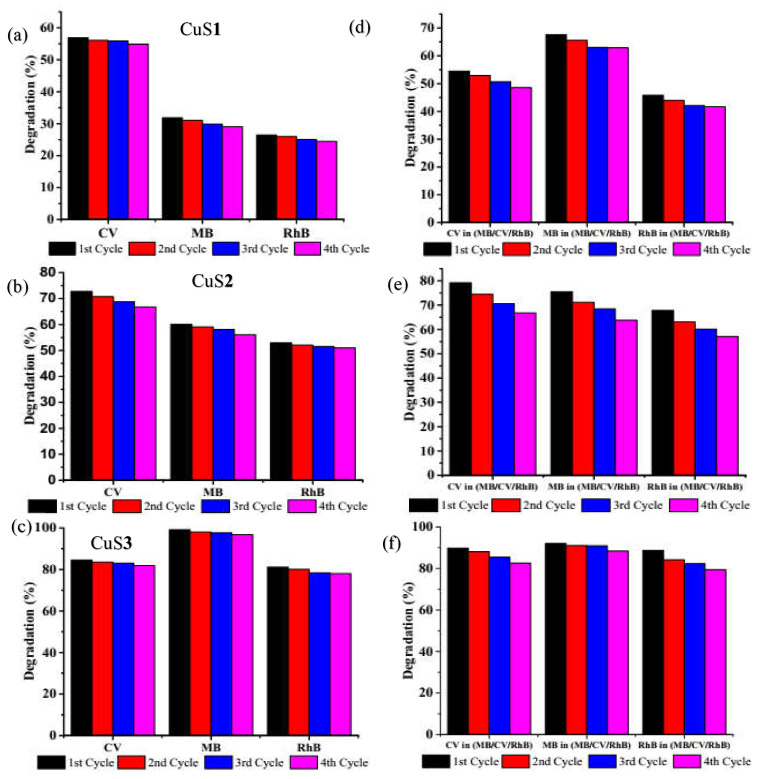
Degradation of crystal violet, methylene blue, and rhodamine B (**a**–**c**) and ternary dyes (**d**–**f**) cycle runs by CuS nanoparticles under visible light irradiation.

For practical purposes, the reusability and stability of the as-prepared CuS nanoparticles as photocatalyst is an important parameter. The as-prepared CuS was evaluated for stability by subjection to four consecutive experimental cycles under the same conditions. After each cycle, the catalyst was washed with distilled water, followed by methanol, filtered, and dried at 70 °C for 2 h before the next cycle. Figure 9 shows the as-prepared CuS nanoparticles show slight variation in degradation efficiencies till the 4th cycle. After four cycles in the single dye, the degradation efficiency was 54%, 30%, and 24% for CV, MB, and RhB respectively over CuS**1**, while over CuS**2**, the degradation efficiency was 64% (CV), 56% (MB) and 50% (RhB). After four cycles, CuS**3** showed degradation efficiency of 82%, 98%, and 80% for CV, MB, and RhB, respectively. The as-prepared CuS nanoparticles also showed good reusability and stability for the degradation of the ternary dyes. With the ternary dyes, degradation efficiencies after four cycles were 54%, 65%, and 82% for CuS**1**, CuS**2,** and CuS**3** respectively for CV while MB degradation efficiencies are 64%, 62%, and 88% and RhB degradation efficiencies are 43% for CuS**1**, 65% for CuS**2** and 78% for CuS**3**. The reduction in degradation efficiency might be due to coverage of the active catalyst sites by the degradation products during reaction and reduction of the catalyst during the filtration process [68]. In addition, the XRD patterns presented in Figure 1 also indicate that the catalyst was not photo corroded as no shift was observed in the diffraction patterns after usage.

### 3.8. Effect of pH

**Figure 10:**
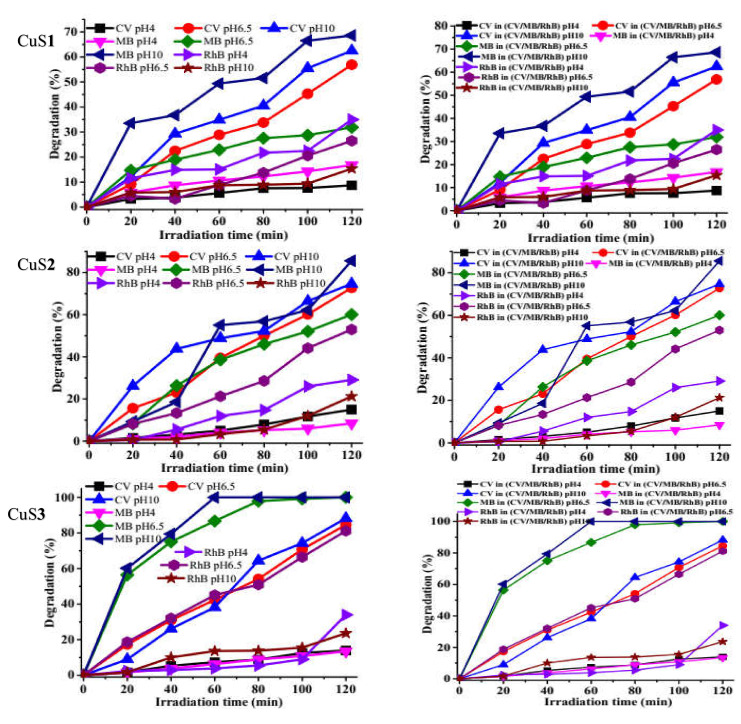
Effect of pH on single dye and corresponding ternary dye solution over CuS nanoparticles.

In the photocatalytic process, the solution pH is an important parameter. The pH of the solution was adjusted by the addition of 0.1M HCl or 0.1M NaOH. The dyes were studied in an acidic medium (pH 3), neutral (6.5), and basic (10). Figure 10 shows the effect of pH on single organic dye and ternary dyes. The results show that CV and MB degradation efficiency decreased in the acidic medium while increasing efficiency in the basic medium. This indicates that H^+^ and OH^−^ are highly involved in the photocatalytic process. There was an increase in H+ concentration in the acidic medium while OH^−^ concentration increased in the basic medium. In the basic medium, OH^−^ reacts with H^+^ to form •OH^−^, which stimulates the degradation process [69,70]. In addition, at lower pH, the surface of the photocatalyst is positively charged; therefore, adsorption of cationic dyes will be low due to repulsive forces hence the reduction of degradation efficiency in the acidic medium [71,72]. On the other hand, there was a drastic decrease in rhodamine B degradation in the basic medium from 26.5%, 52.9%, and 81.1% for CuS**1**, CuS**2,** and CuS**3** to 15.4%, 22.2%, and 23.6%. The decrease observed in the basic medium might be due to RhB being in the zwitterionic (deprotonated carboxylic function of RhB), and dimeric form, which is negatively charged, and the photocatalyst surface is also negatively charged. Thus it creates repulsive forces [73]. In addition, low degradation efficiency was observed in an acidic medium, indicating that neutral pH is favorable for RhB degradation. The same trend was observed in the degradation of the ternary dye solution.

### 3.9. Identification of the Photocatalytic Degradation Products

**Scheme 2:**
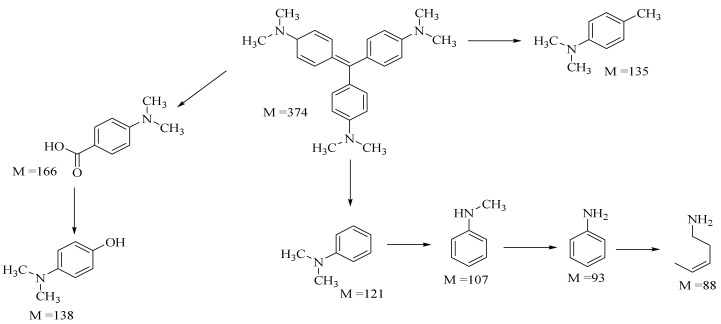
Degradation pathway of crystal violet over CuS nanoparticles.

**Scheme 3:**
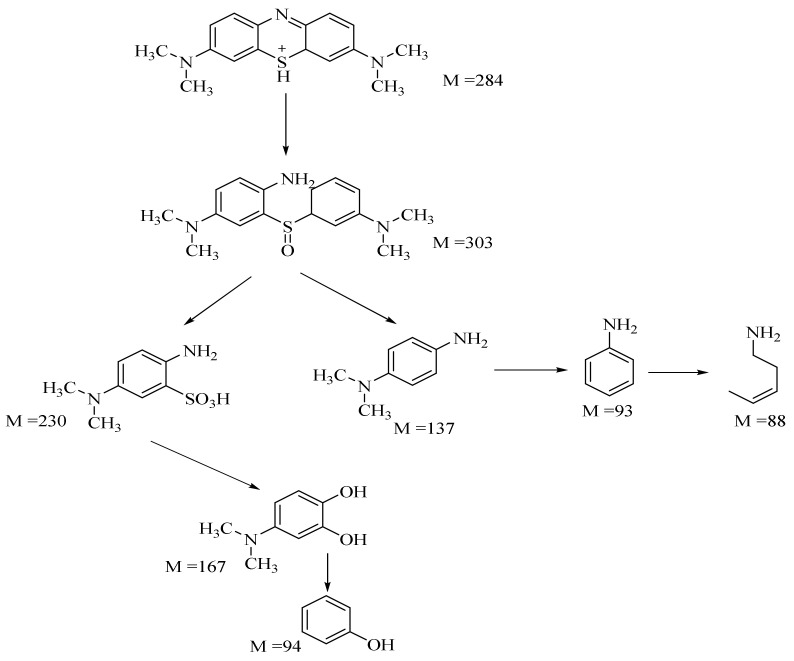
Degradation pathway of methylene blue over CuS nanoparticles.

LC-MS was used to identify the photocatalytic degradation products of the dyes as shown in Scheme 2 and Scheme 3 (Figures S9–S12). Six degraded products identified from CV dye were N-methyl aniline, N, N-dimethyl aniline, 4-methyl-N, N-dimethylaniline, 4-dimethylamino phenol, 4-dimethylamino benzoic acid, and pent-3-en-1-amine.

These products were similar to those reported in the literature [74,75]. The formation of these products shows that hydroxyl radicals are the main species involved in the photocatalytic process, as confirmed by the scavenging activity. The degradation pathway is given in Scheme 2. The intermediate and final product of methylene blue degradation was also analyzed. The fragment ions observed in the mass spectra were identified according to the literature [76] as presented in Scheme 3. In rhodamine B, the rhodamine B peak intensity was reduced at the final stage to form the zwitterionic form of rhodamine B.

## 4. Conclusions

CuS nanoparticles were prepared from Cu(II) dithiocarbamate single-source precursor at three different reaction times to study the effect of reaction time on the structural and optical properties of the as-prepared copper sulfide nanoparticles. HRTEM images revealed mixtures of spherical, hexagonal, and rod-shaped copper sulfide nanoparticles. At 30 min, CuS**1** with an average particle size of 31.47 ± 0.85 nm was obtained, while at 1 h, CuS**2** with a particle size of 21.59 ± 0.68 nm was obtained, and CuS**3** was prepared at 2 h with average particles size of 17.77 ± 6.26 was obtained. The HRTEM images showed that as thermolysis time increases, the average particle size of the as-prepared CuS reduces. Powder X-ray diffraction patterns confirmed hexagonal covellite CuS crystalline phase for the nanoparticles irrespective of thermolysis time. The EDX spectra and elemental mapping confirmed homogeneous distributions of copper and sulfur in the as-prepared copper sulfide nanoparticles. FTIR spectra confirmed octadecylamine bond to the surface of the as-prepared CuS nanoparticles. The photocatalytic degradation of crystal violet (CV), methylene blue (MB), rhodamine B (RhB), and the ternary mixture of the three dyes (CV/MB/RhB) were evaluated. The results showed that CuS**3** showed high photocatalytic degradation efficiency for the degradation of CV (84%), MB (100%), and RhB (81%). It also degraded the ternary mixture of the three dyes. This could be ascribed to its small particle size, low recombination electron-hole rate, copper vacancies, and surface defects. The role of pH and scavengers on the photocatalytic degradation efficiency was also studied with ^•^O_2_^−^_,_ h^+^ and ^•^OH found to be the dominant active species in the photodegradation process. The as-prepared CuS nano photocatalysts are highly stable and recyclable for the photocatalytic degradation of the organic dyes. The stability and recyclability of the as-prepared CuS make them useful as potential photocatalysts for removing CV, MB, and RhB in industrial wastewater.

## Figures and Tables

**Table 1:** Degradation of organic dyes using copper sulfide as photocatalyst.

Dyes	Irradiation Time (min)	Light Source (lamp)	Degradation (%)	Reference
Methylene blue	400	25 W daylight	85.38	[56]
	180	36 W daylight	92	[57]
	80	Xenon	52.39	[58]
	40	500 W Xenon	7.2	[19]
	120	70 W Mercury	100	This work
Crystal violet	60	70 W Mercury	6	[59]
	120	200 W Tungsten	48.1	[60]
	120	70 W Mercury	84.6	This work
Rhodamine B	160	500 W Halogen	24.23	[61]
	120	15 W Tungsten	42	[62]
	60	300 W Xenon	60	[63]
	120	70 W Mercury	81.4	This work
CV in (CV/MB/RhB)	120	70 W Mercury	89.8	This work
MB in (CV/MB/RhB)			92.1	
RhB in (CV/MB/RhB)			88.7	

**Table 2:** The photocatalytic degradation parameters of single and mixed dye over CuS nanoparticles under visible light irradiation.

Dye	Catalyst	Degradation Efficiency (%)	Rate Constant (min^−1^)	R^2^
CV	CuS**1**	56.9	0.0066	0.9674
	CuS**2**	72.8	0.0104	0.9794
	CuS**3**	84.6	0.0145	0.9621
MB	CuS**1**	31.9	0.006	0.9461
	CuS**2**	60.1	0.0078	0.9922
	CuS**3**	99.2	0.0481	0.9608
RhB	CuS**1**	26.5	0.0025	0.9263
	CuS**2**	53.0	0.0062	0.9415
	CuS**3**	81.1	0.0127	0.9354
CV in (CV/MB/RhB)	CuS**1**	545	0.0062	0.9888
	CuS**2**	79.2	0.0126	0.9794
	CuS**3**	89.8	00166	0.9192
MB in (CV/MB/RhB)	CuS**1**	67.6	0.0093	0.9527
	CuS**2**	75.5	0.0122	0.9900
	CuS**3**	92.1	0.0197	0.9715
RhB in (CV/MB/RhB)	CuS**1**	45.8	0.0048	0.9684
	CuS**2**	67.8	0.0092	0.9925
	CuS**3**	88.7	0.0164	0.9561

## Supplementary Materials

The following are available online at https://www.mdpi.com/article/10.3390/nano11082000/s1, Figure S1: SEM images and EDX spectra with elemental mappings of CuS**1**, CuS**2** and CuS**3** nanoparticles; Figure S2: FTIR spectra overlay of octadecylamine and CuS nanoparticles prepared at different reaction time; Figure S3: Absorption spectra of crystal violet degradation over CuS nanoparticles; Figure S4: Absorption spectra of methylene blue degradation over CuS nanoparticles; Figure S5: Absorption spectra of rhodamine B degradation over CuS nanoparticles; Figure S6: Absorption spectra of CV/MB/RhB degradation over CuS**1** nanoparticles; Figure S7: Absorption spectra of CV/MB/RhB degradation over CuS**2** nanoparticles; Figure S8. Absorption spectra of CV/MB/RhB degradation over CuS3 nanoparticles; Figure S9: Absorption spectra of CV/MB/RhB degradation over CuS**3** nanoparticles; Figure S10: LC-MS ESI of crystal violet at initial (before photocatalysis) and final (after photocatalysis); Figure S11: LC-MS ESI of rhodamine B at initial (before photocatalysis) and final (after photocatalysis), Figure S12: LC-MS ESI of ternary mixed dye at initial (before photocatalysis) and final (after photocatalysis).
