# Supplementary material for: Enhanced Photocatalytic Degradation of Ternary Dyes by Copper Sulfide Nanoparticles
Source: Nanomaterials (Basel). 2021 Aug 4;11(8):2000. doi: 10.3390/nano11082000 (PMC8398049; doi:10.3390/nano11082000)
Supplement: Supplementary file 1 [file nanomaterials-11-02000-s001.zip › nanomaterials-1320320-supplementary.pdf]

Enhanced Photocatalytic Degradation of Ternary Dyes by Copper Sulfide Nanoparticles

Peter A. Ajibade * and Abimbola E. Oluwalana

School of Chemistry and Physics, University of KwaZulu-Natal, Private Bag X01, Scottsville, Pietermaritzburg 3209, South Africa; 217075609@stu.ukzn.ac.za
* Correspondence: ajibadep@ukzn.ac.za

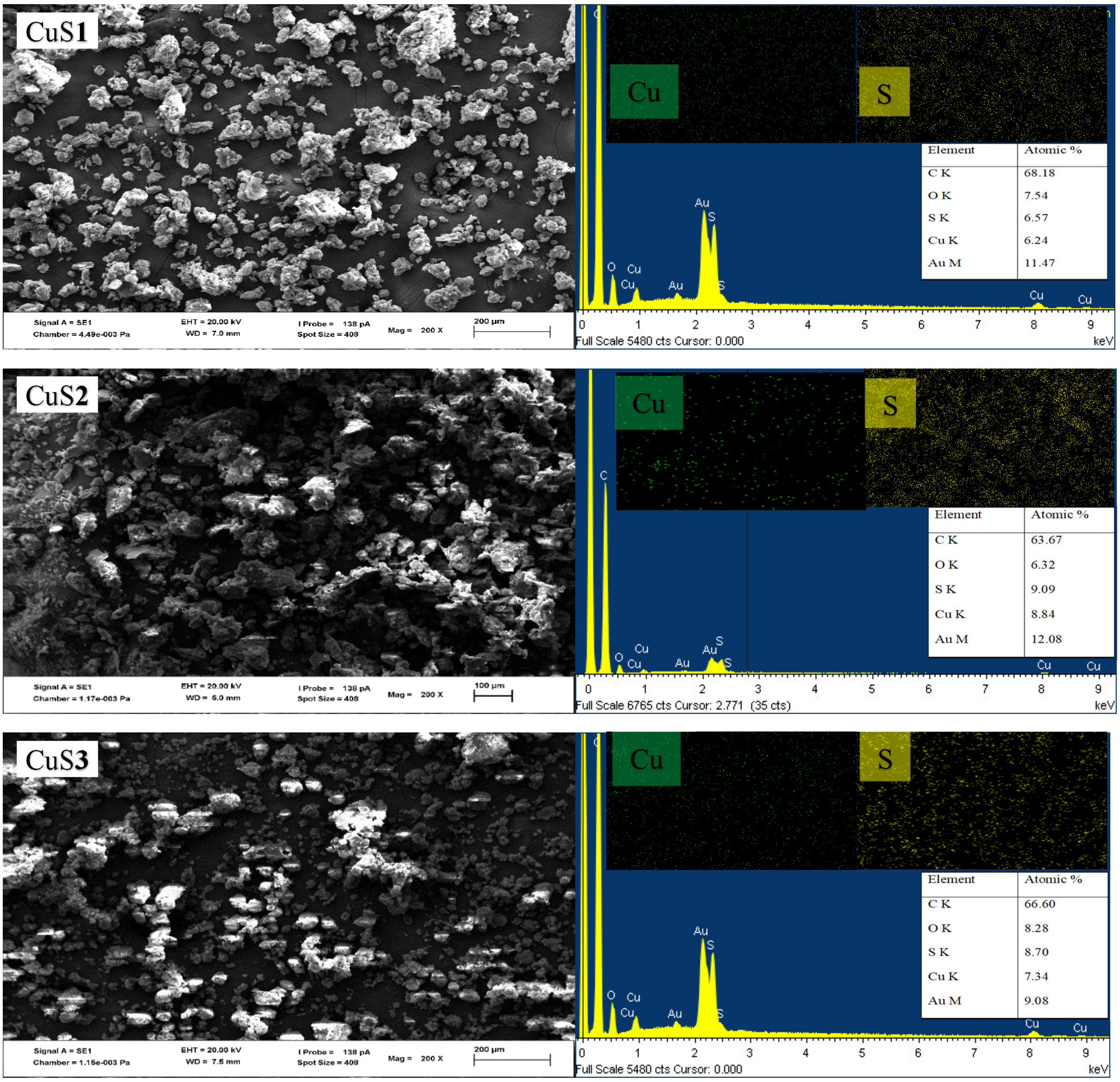

Figure S1. SEM images and EDX spectra with elemental mappings of CuS**1**, CuS**2** and CuS**3** nanoparticles.

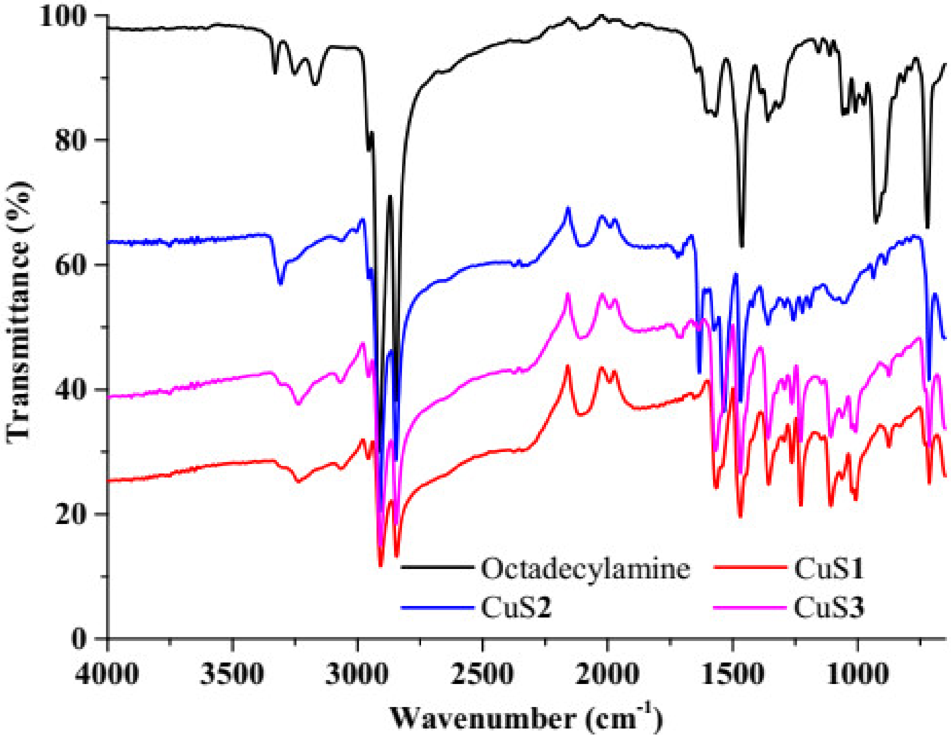

Figure S2. FTIR spectra overlay of octadecylamine and CuS nanoparticles prepared at different reaction time.

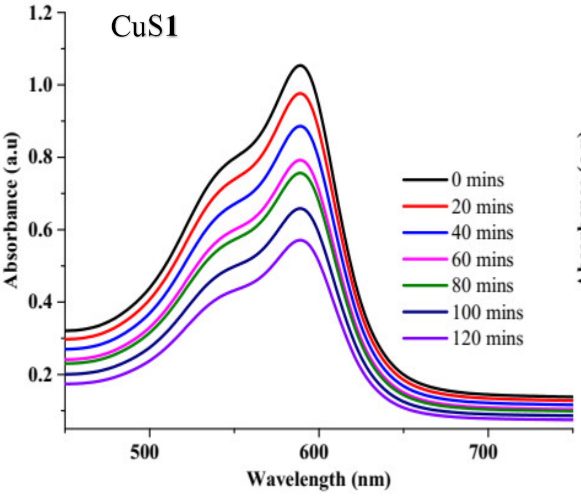

(a)

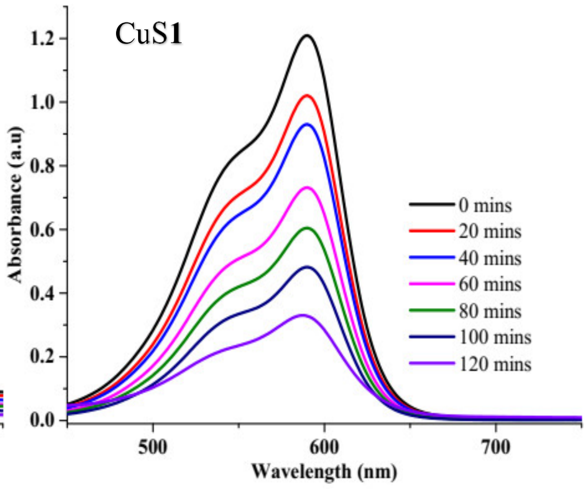

(b)

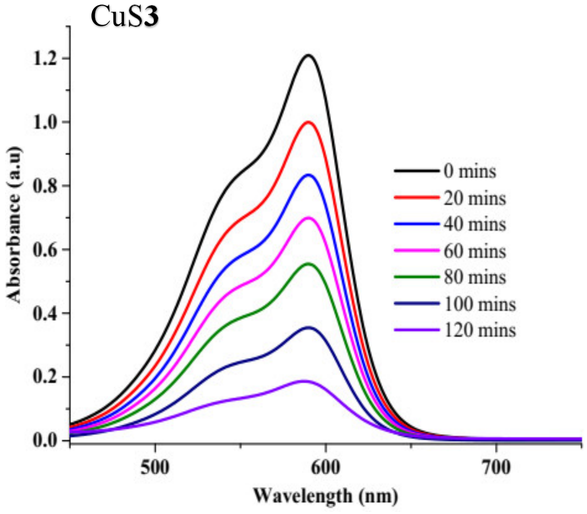

(c)

Figure S3. Absorption spectra of crystal violet degradation over CuS (**a**)CuS**1** (**b**)CuS**2** (**c**)CuS**3** nanoparticles.

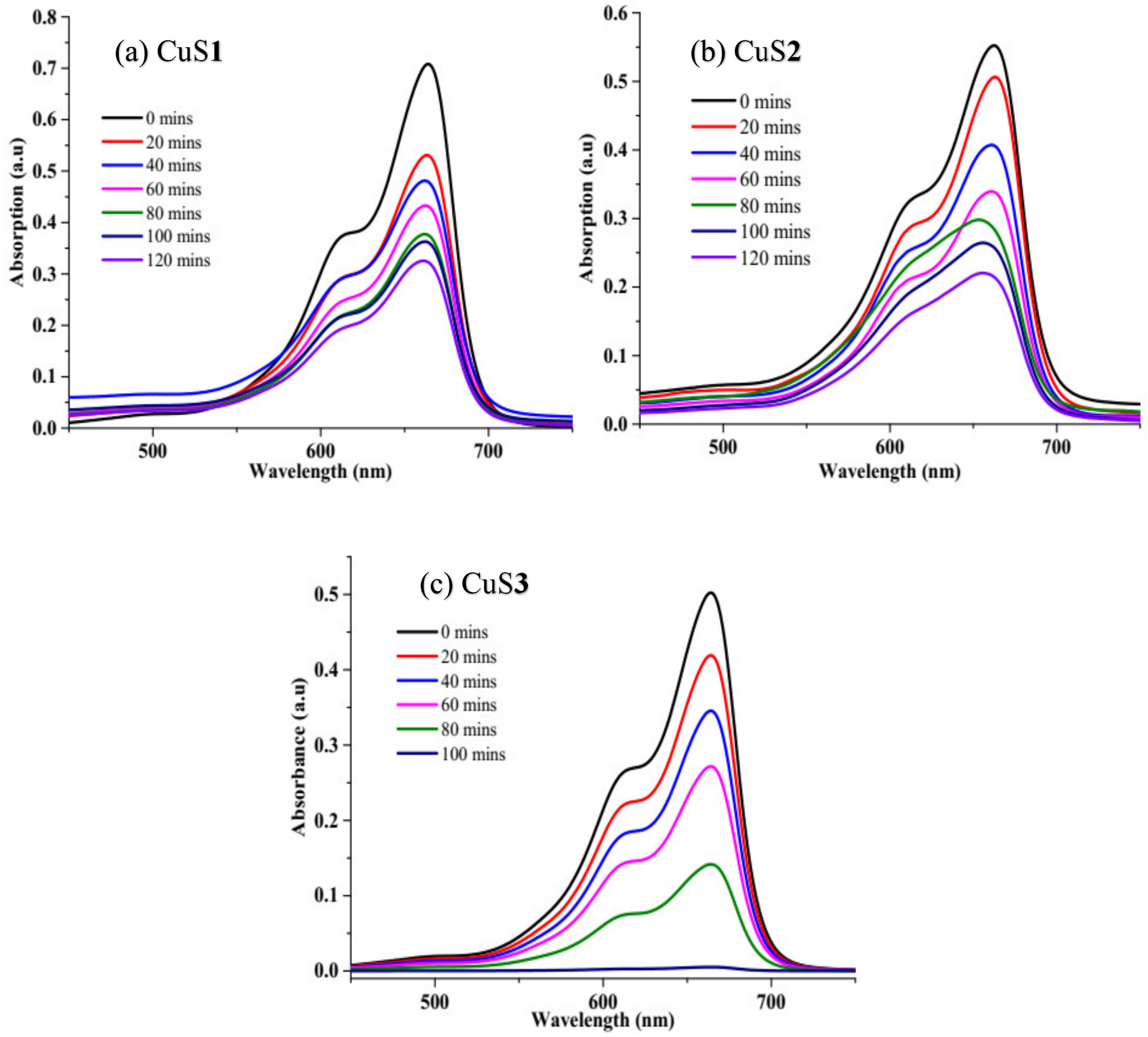

Figure S4. Absorption spectra of methylene blue degradation over CuS (**a**)CuS**1** (**b**)CuS**2** (**c**)CuS**3** nanoparticles.

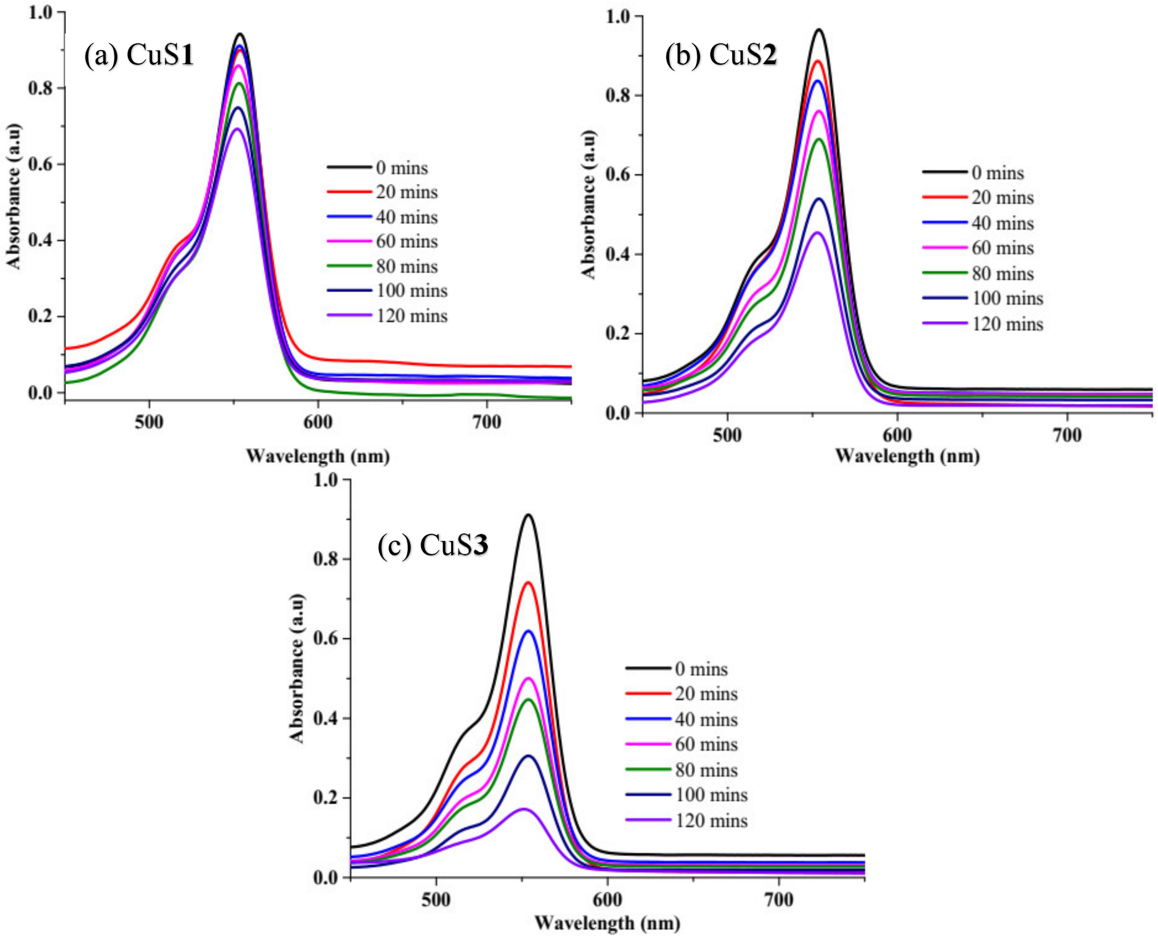

Figure S5. Absorption spectra of rhodamine B degradation over CuS (**a**)CuS**1** (**b**)CuS**2** (**c**)CuS**3** nanoparticles.

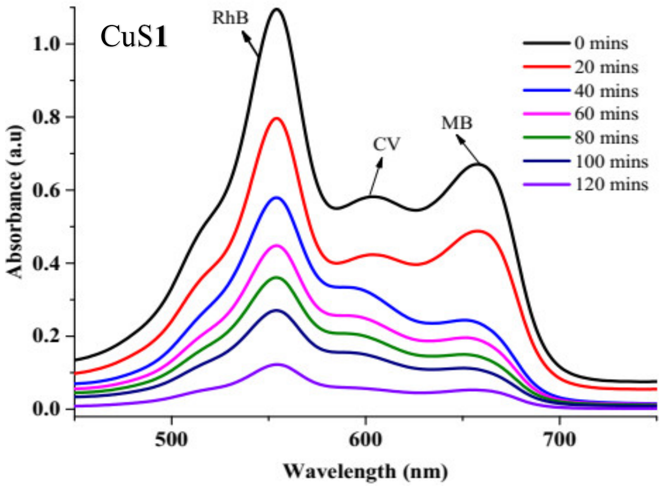

Figure S6. Absorption spectra of CV/MB/RhB degradation over CuS**1** nanoparticles.

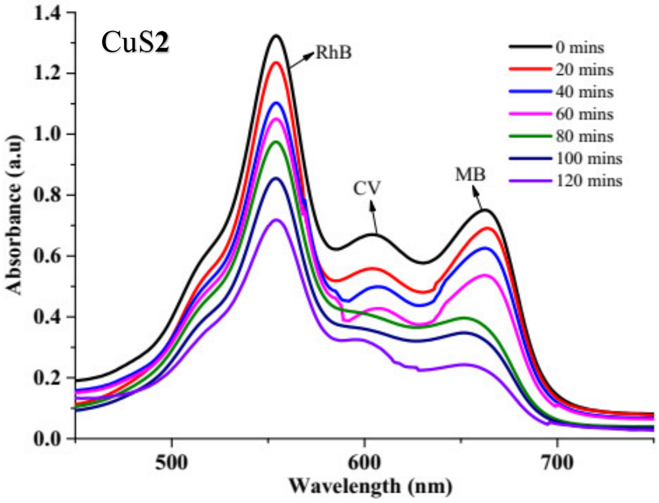

Figure S7. Absorption spectra of CV/MB/RhB degradation over CuS**2** nanoparticles.

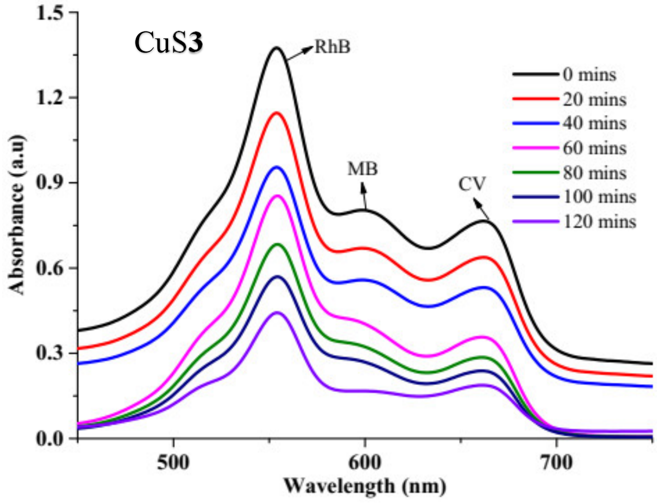

Figure S8. Absorption spectra of CV/MB/RhB degradation over CuS**3** nanoparticles.

(a)

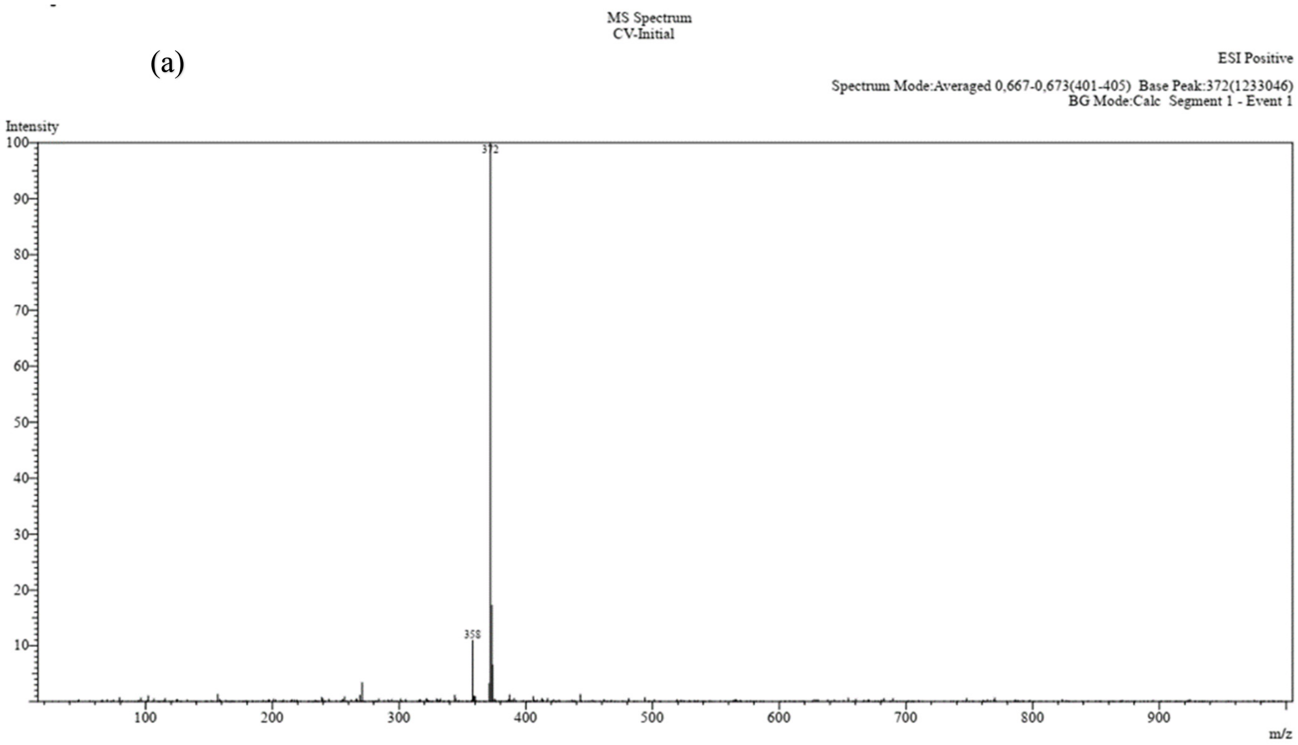

(b)

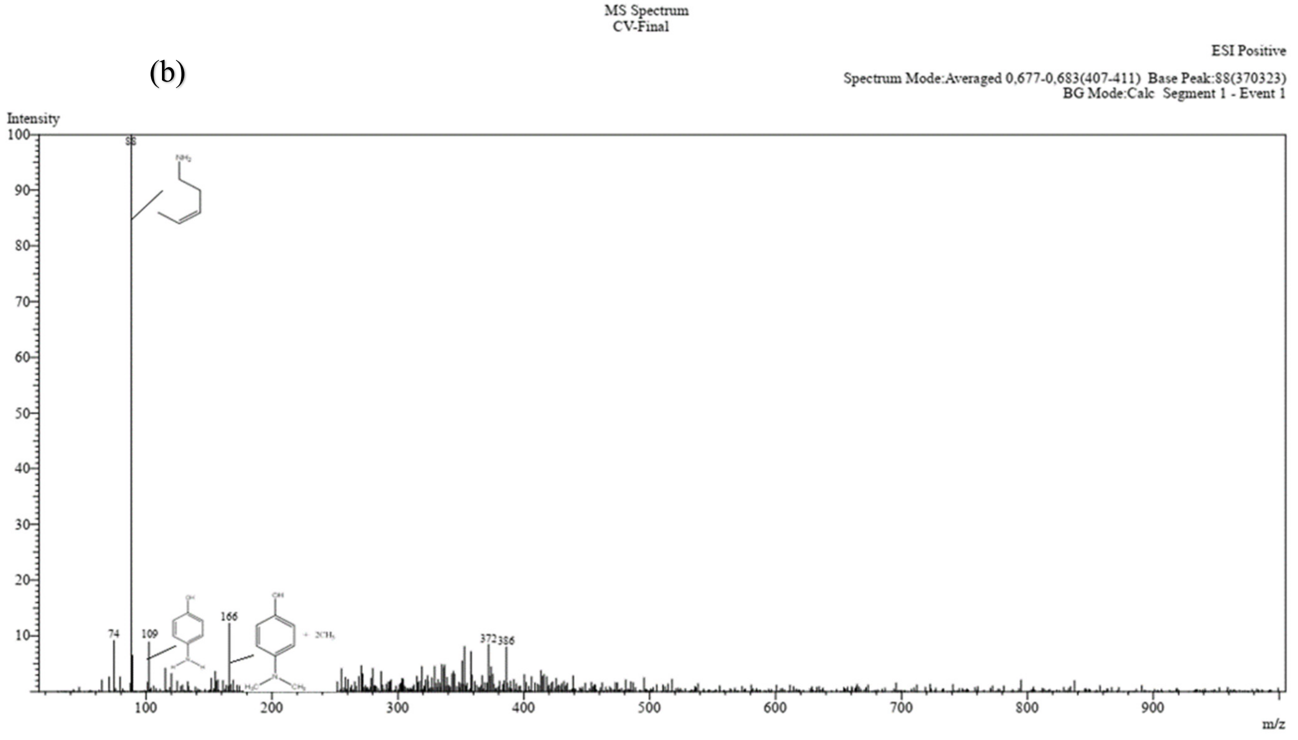

Figure S9. LC-MS ESI of crystal violet at initial (**a**) and final (**b**).

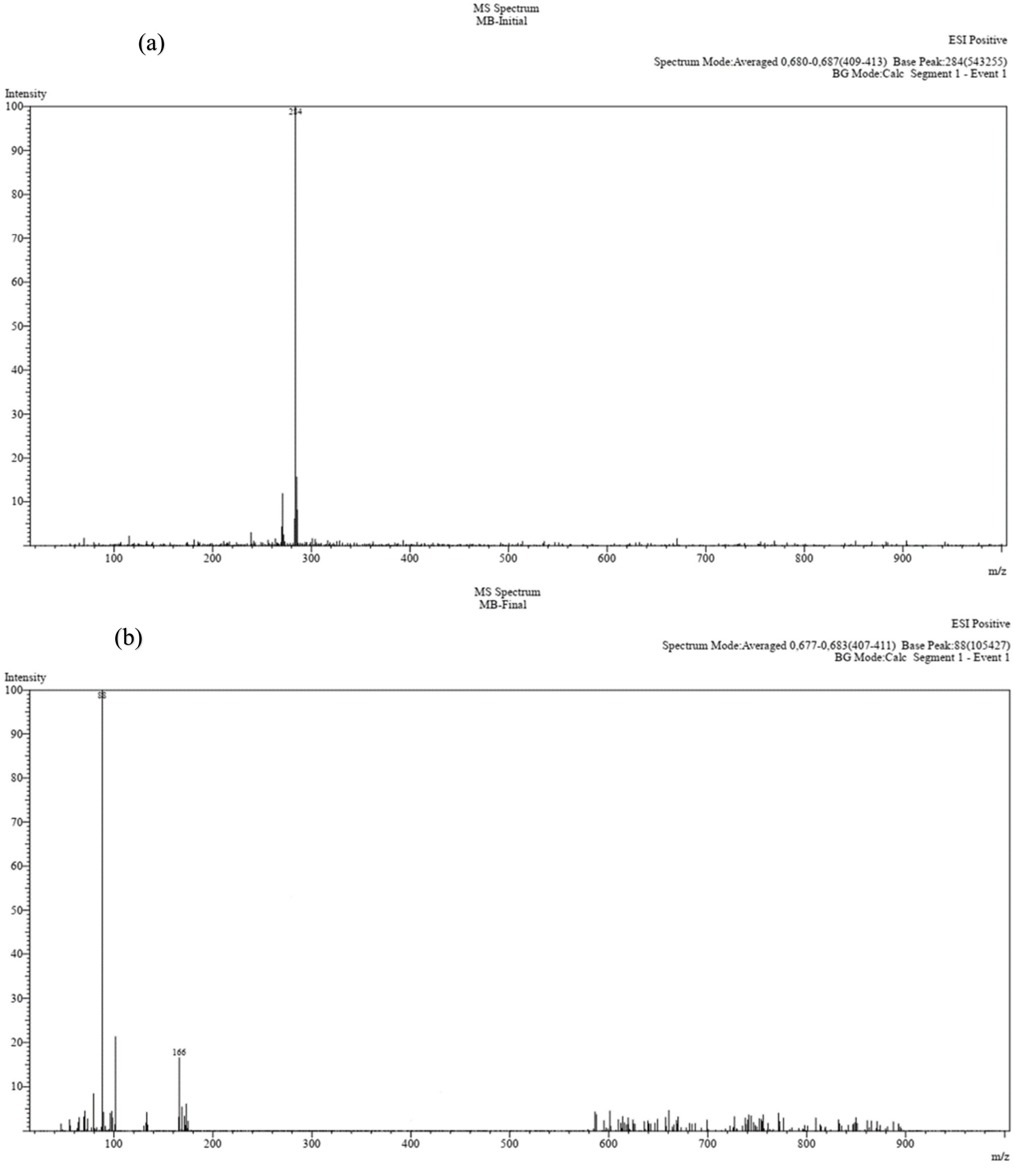

Figure S10. LC-MS ESI of methylene blue at initial (**a**) and final (**b**).

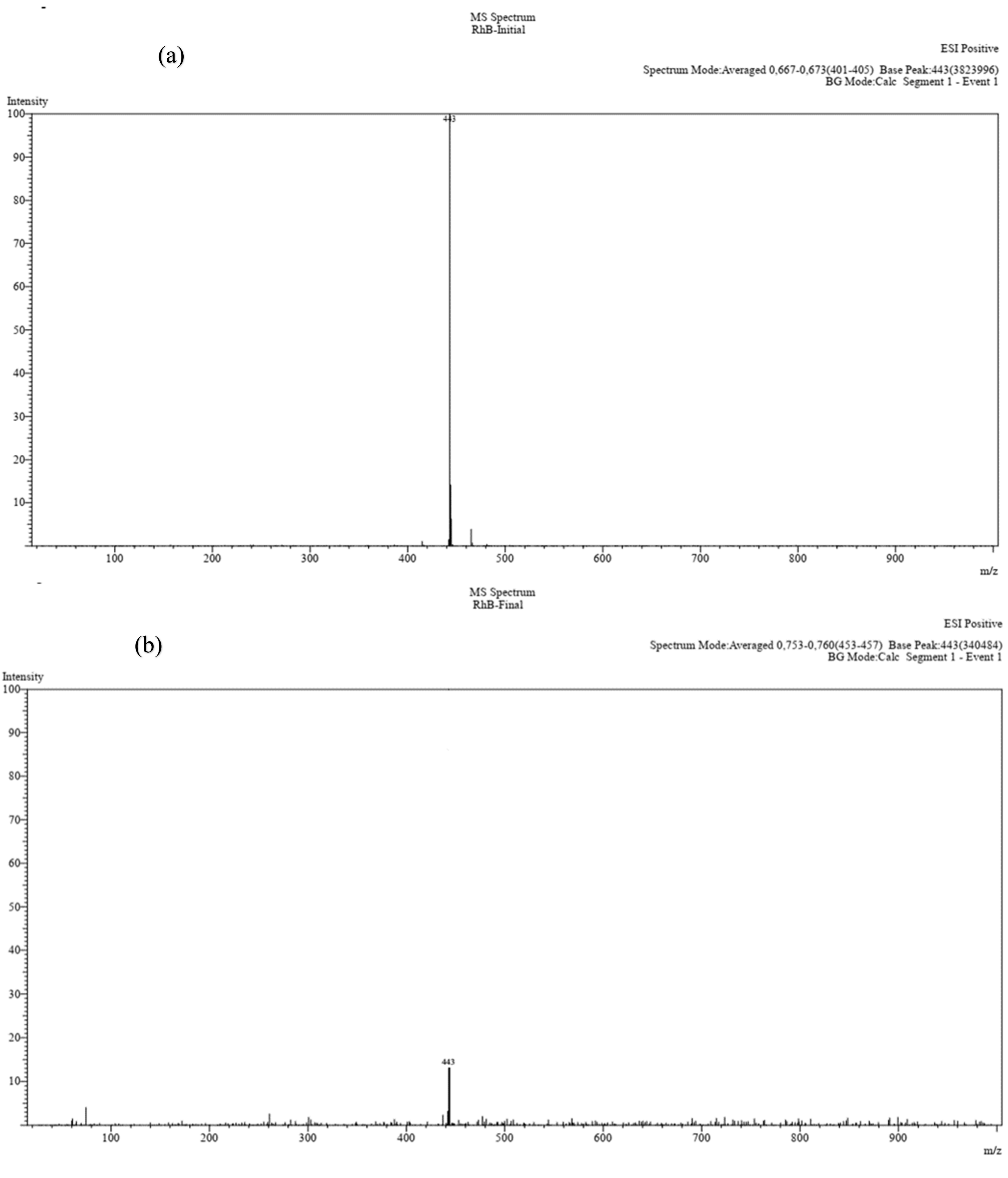

Figure S11. LC-MS ESI of rhodamine B at initial (**a**) and final (**b**).

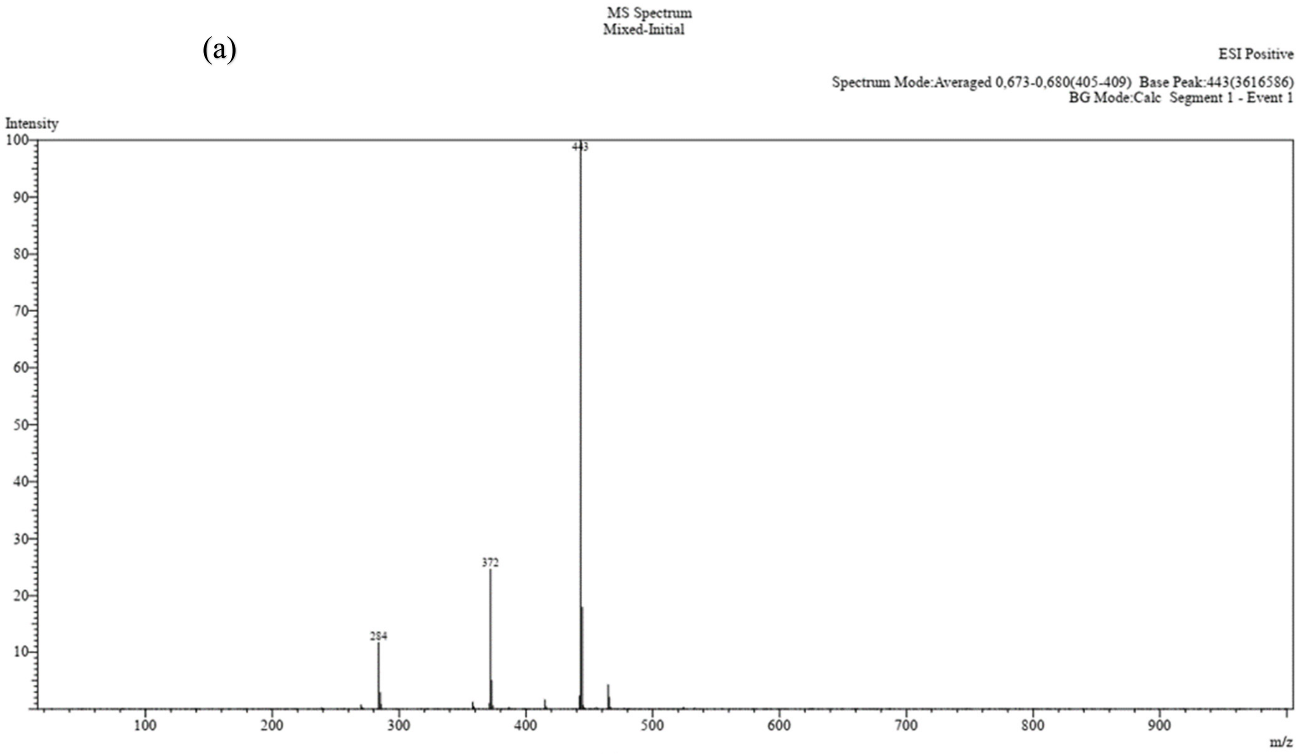

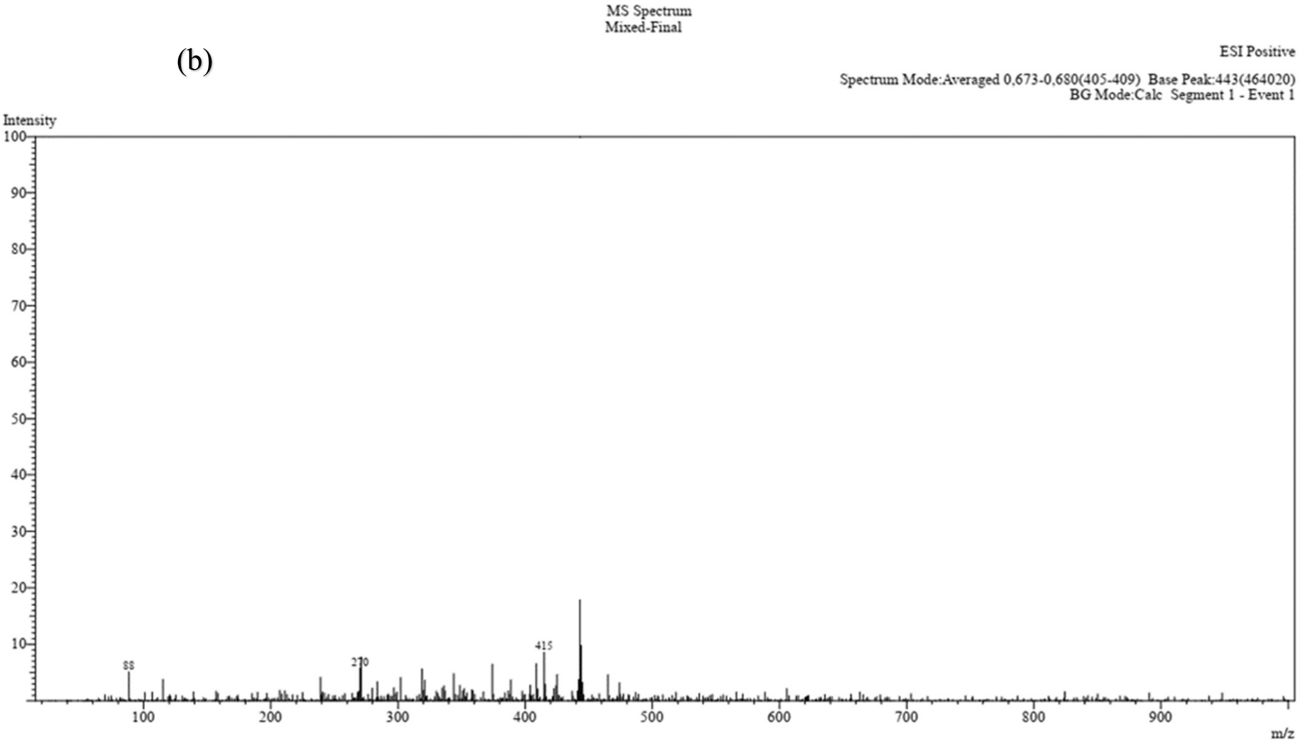

Figure S12. LC-MS ESI of ternary mixed dye at initial (**a**) and final (**b**).
